# Clinical reappraisal of the composite international diagnostic interview version 3.3 in Qatar's National Mental Health Study

**DOI:** 10.1002/mpr.2013

**Published:** 2024-05-10

**Authors:** Salma M. Khaled, Iman Amro, Menatalla Abdelkader, Dalia Al Bahari, Mahmoud Al Shawwaf, Majid Alabdulla, Ahmed Alhassan, Amal Ali, Sheeren Aly, Asmaa Amin, Wai Tat Chiu, James Currie, Hana El Fakki, Michael B. First, Mohammed H. O. Hassan, Zainab Hijawi, Rumaisa Mohammed, Marwa Nofal, Salma Salman, Nancy A. Sampson, Peter W. Woodruff, Ronald C. Kessler

**Affiliations:** ^1^ Department of Population Medicine College of Medicine Qatar University Doha Qatar; ^2^ The Social & Economic Survey Research Institute Qatar University Doha Qatar; ^3^ Qatar Hamad Medical Corporation Doha Qatar; ^4^ College of Medicine, Qatar University Doha Qatar; ^5^ Department of Health Care Policy Harvard Medical School Boston Massachusetts USA; ^6^ Columbia University Department of Psychiatry New York New York USA; ^7^ School of Medicine and Population Health University of Sheffield Sheffield UK

**Keywords:** clinical reappraisal, composite international diagnostic interview (CIDI), diagnostic and statistical manual version 5 (DSM‐5), diagnostic assessment, epidemiology, validity

## Abstract

**Objectives:**

Lifetime DSM‐5 diagnoses generated by the lay‐administered Composite International Diagnostic Interview for DSM‐5 (CIDI) in the World Mental Health Qatar (WMHQ) study were compared to diagnoses based on blinded clinician‐administered reappraisal interviews.

**Methods:**

Telephone follow‐up interviews used the non‐patient edition of the Structured Clinician Interview for DSM‐5 (SCID) oversampling respondents who screened positive for five diagnoses in the CIDI: major depressive episode, mania/hypomania, panic disorder, generalized anxiety disorder, and obsessive‐compulsive disorder. Concordance was also examined for a diagnoses of post‐traumatic stress disorder based on a short‐form versus full version of the PTSD Checklist for DSM‐5 (PCL‐5).

**Results:**

Initial CIDI prevalence estimates differed significantly from the SCID for most diagnoses ($\chi_{1}^{2}$ = 6.6–31.4, *p* = 0.010 < 0.001), but recalibration reduced most of these differences and led to consistent increases in individual‐level concordance (AU‐ROC) from 0.53–0.76 to 0.67–0.81. Recalibration of the short‐form PCL‐5 removed an initially significant difference in PTSD prevalence with the full PCL‐5 (from $\chi_{1}^{2}$ = 610.5, *p* < 0.001 to $\chi_{1}^{2}$ = 2.5, *p* = 0.110) while also increasing AU‐ROC from 0.76 to 0.81.

**Conclusions:**

Recalibration resulted in valid diagnoses of common mental disorders in the Qatar National Mental Health Survey, but with inflated prevalence estimates for some disorders that need to be considered when interpreting results.

## 1 INTRODUCTION

We present a series of disorder‐specific analyses comparing diagnoses of lifetime DSM‐5 disorders based on the Composite International Diagnostic Interview or the CIDI (Kessler & Üstün, 2004a) with diagnoses of the same individuals based on a follow‐up interview with the DSM‐5 version of the Structured Clinical Interview for DSM‐5 (First et al., 2015) in the World Mental Health Qatar (WMHQ) study. The WMHQ is the first nationally representative epidemiological survey of mental disorders conducted in Qatar (Khaled, Al‐Abdulla, et al., 2023; Khaled et al., 2021). The WMHQ was carried out in conjunction with the WMH Survey Initiative (Scott et al., 2018). The CIDI is the lay‐administered diagnostic interview used in all WMH surveys.

Developed initially to operationalize DSM‐IV criteria, the CIDI was recently updated to operationalize DSM‐5 criteria. This more recent version or the CIDI version 3.3 is the one used in the WMHQ. The SCID, in comparison, is the gold standard semi‐structured research diagnostic interview that has been used in prior WMH CIDI clinical reappraisal studies in the US (Kessler et al., 2006), Latin America (Montoya Gonzalez et al., 2016), Europe (Alonso et al., 2004; Haro et al., 2006; Kessler et al., 2008), Asia (Ghimire et al., 2013; Lu et al., 2015), and the Middle East (Kessler et al., 2020). These calibration studies were carried out to make sure the diagnostic classifications and thresholds used in the WMH surveys are consistent with those based on clinical assessments. The SCID was used in these clinical reappraisal studies to reinterview a subsample of CIDI respondents, oversampling those who met each CIDI diagnosis of central interest in addition to a subsample of respondents who met criteria for none of these disorders in the CIDI. These clinical reappraisal samples were weighted to adjust for the over‐sampling of CIDI cases. SCID interviewers were blinded to CIDI diagnoses. The same approach was used in the WMHQ clinical reappraisal study. Although results of previous WMH CIDI clinical calibration studies showed that diagnoses based on the CIDI had generally good concordance with independent diagnoses based on the SCID, these earlier studies were based on DSM‐IV diagnoses. The WMHQ clinical reappraisal study is the first one to validate the CIDI for DSM‐5. It is noteworthy that the WMH version of CIDI was developed specifically to optimize diagnostic validity in community surveys based on insights gained from clinical reappraisal studies carried out with earlier versions of CIDI (Kessler et al., 1998; Wittchen et al., 1995, 1996) as well as insights gained from the methodological literature in best practices for designing community surveys (Converse & Presser, 1986; Presser et al., 2004).

Central to the WMH CIDI is the fact that all diagnostic assessments use a stem‐branch structure that focuses initially on recent (past 30 days) disorder‐specific symptoms and then uses cognitive psychological strategies to motivate respondents to engage in active memory search and to facilitate this process in answering questions about prior lifetime episodes and symptoms within these episodes. Subsequent disorder‐specific questions are then used to distinguish true positives from subthreshold cases. Questions about course of illness are then administered to obtain basic information about age‐of‐onset, age‐of‐recency, and number of years in episode between age‐of‐onset and age‐of‐recency. More details about these question sequences are described elsewhere (Kessler et al., 2020).

Past CIDI clinical reappraisal studies showed that a major reason for discrepancies between lifetime diagnoses based on the CIDI and the SCID involved instances in which CIDI diagnoses of lifetime prevalence among remitted cases were classified in the SCID as not meeting lifetime criteria rather than having subthreshold episodes (Kessler & Üstün, 2004b). Debriefing suggested that this discrepancy occurred because survey respondents became aware of the stem‐branch structure of the CIDI and recognized that they could shorten the follow‐up interview by denying the diagnostic stem questions in the clinical reappraisal follow‐up interview. This observation is especially critical given that clinical interviewers are more used to assessing patients who present to them with current problems than probing for past lifetime occurrences of remitted disorders (Edelbrock et al., 1999; Kessler & Üstün, 2008).

As discussed in more detail elsewhere (Haro et al., 2006; Kessler et al., 2018), we addressed this tendency for respondents to deny questions in the WMH clinical reappraisal studies by partially unblinding clinical interviewers to respondent endorsement of diagnostic stem questions. That is, we focused on the subset of WMH survey respondents who reported in the CIDI that they sometime in their life experienced one or more of the core criteria for the disorder under investigation. For example, in the case of major depressive episode, having a time lasting 2 weeks or longer when most of the day nearly every day they experienced dysphoria or anhedonia, we told the clinical interviewer that the respondent reported this experience in the CIDI. The clinical interviewer then began the assessment of that disorder by informing the respondent that the interview would now focus on one of the things the respondent reported in their earlier interview, repeating the diagnostic stem question(s) that the respondent endorsed in the earlier CIDI interview, and then probing that positive response for more detailed information designed to distinguish between true cases and non‐cases.

A concern might be raised that this partial unblinding would bias results, but this possibility is taken into consideration in all WMH clinical reappraisal studies by enriching the clinical reappraisal sample for respondents who screened positive on the diagnoses but failed to meet CIDI diagnostic criteria. Importantly, as detailed below, substantial proportions of respondents in the WMHQ (as in community epidemiological surveys elsewhere in the world) endorsed diagnostic stem questions but failed to meet full criteria for disorders. The real challenge for clinical interviewers given this fact is to distinguish screened positives who meet full diagnostic criteria from those that do not. To meet this challenge became the focus of our clinical reappraisal interviews. We recognize that the design of the stem questions leaves open the possibility of under‐diagnosis due to the CIDI screens missing some people who met lifetime diagnostic criteria but whose past episodes were not reported. Therefore, we developed CIDI screens to address this possibility by using memory priming strategies to maximize the proportion of true cases that endorse a diagnostic stem question. Nonetheless, the prevalence estimates in the WMHQ should be considered conservative because of the possibility of residual false negatives.

The diagnoses assessed in the WMHQ CIDI‐SCID clinical reappraisal study were major depressive episode, mania/hypomania, panic disorder, generalized anxiety disorder, and obsessive‐compulsive disorder. We customized the SCID to focus on these five disorders for purposes of this study. Details of the SCID interview customization process are described elsewhere (Amro et al., 2023). The interview structure allowed the interviewers to assess respondents' current psychopathology and history and then to assess each module of the five diagnoses. Active memory search priming strategies were used to probe for recall of lifetime episodes among respondents who denied lifetime histories in initial questioning. Follow‐up questions were then asked to distinguish true positive from false positive cases among those who endorsed diagnostic stem questions. The interviewers used the SCID probing techniques to elaborate on symptom reports before making ratings.

In addition to the CIDI‐SCID calibration exercise, we examined concordance between diagnoses based on a 6‐question short‐form of the 20‐question PTSD Checklist adapted for DSM‐5 (PCL‐5) that was administered to all respondents in the WMHQ (Lang & Stein, 2005) and diagnoses based on the full 20‐item PCL‐5 (Weathers et al., 2013). The PCL‐5 is a validated fully‐structured assessment of PTSD (Bovin et al., 2016) that is widely‐used in both clinical and research settings. The 6‐item short‐form was used rather than the full PCL‐5 to assess both lifetime and recent prevalence based on evidence that short‐forms can accurately reproduce diagnoses based on the full PCL‐5 (Zuromski et al., 2019). However, as there is no guarantee that the same diagnostic validity is true in the Qatari population, in the WMHQ we administered the full 20‐question PCL‐5 to a random 10% of the sample of respondents to assess lifetime PTSD. This allowed a test to be made of whether the original calibration is accurate in Qatar and, if not, to recalibrate and reproduce diagnoses based on the full PCL‐5.

## 2 METHODS

**FIGURE 1:**
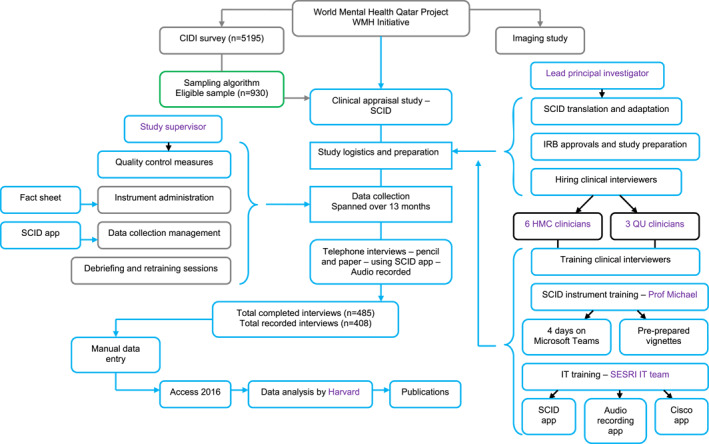
Overview of methodology used in the Qatar‐based clinical reappraisal study of the composite international diagnostic interview (CIDI version 3.3).

A detailed description of the methodology used in the WMHQ clinical reappraisal study is available elsewhere (Amro et al., 2023). However, the following section will briefly highlight the main methods related to study sampling, study design, and fielding procedures. Figure 1 provides a map for the WMHQ clinical reappraisal design.

### 2.1 Study sample‐ CIDI survey sample

As detailed in the companion paper, the main WMHQ sampling design is described elsewhere in this issue (Khaled et al., 2023). Briefly, the survey was conducted via telephone interviews and targeted Arabic‐speaking residents of Qatar (Khaled et al., 2023). A stratified multi‐stage sampling procedure was used to draw a representative sample of Qatari nationals and non‐Qatari (Arab) residents for the study sample (Khaled et al., 2023). The survey was directed to male and female respondents aged 18 and above who lived in Qatar during the survey reference period. A cell phone frame suitable for the survey was created by the Social and Economic Survey Research Institute (SESRI) of Qatar University (QU) with the help of the main telecommunication provider in Qatar. As the vast majority (98%) of adults in Qatar have at least one cell phone, the frame was expected to provide excellent coverage for this target population (SESRI Omnibus, 2019). As described in detail elsewhere in this issue, we weighted the sample to account for variation in probabilities of selection, variation in response rates, and post‐stratification calibration (Khaled et al., 2023). As in other WMH surveys, the WMHQ used a two‐part sample in which all respondents. The Part I sample were administered a set of core disorders while a Part II sample made up of all respondents with any Part I disorder plus a probability subsample of other Part I respondents who were administered additional questions about other disorders and correlates. One of the disorders assessed in the clinical reappraisal sample, obsessive‐compulsive disorder, was a Part II disorder. The others were Part I disorders.

### 2.2 Study sub‐sample‐ SCID interview sample

The clinical reappraisal sample was selected using an algorithm that over‐sampled respondents who met criteria in the five CIDI diagnostics modules of interest and roughly equal numbers of respondents who endorsed stem questions for these diagnoses (but did not meet CIDI diagnostic criteria) along with a smaller proportion who did not endorse diagnostic stem questions. The five disorders were major depressive episode, mania/hypomania, generalized anxiety disorder, obsessive‐compulsive disorder, and panic disorder. To achieve precision equivalent to past research that assessed CIDI‐SCID interview concordance, a target of 50 CIDI cases per disorder disease was set (Haro et al., 2006). Full details on the sampling methodology are available elsewhere (Amro et al., 2023).

### 2.3 SCID study design & field procedures

As noted above, the clinical reappraisal interview was an adapted version of the SCID for DSM‐5 (First et al., 2015). As mapped in Figure 1, the SCID was translated and adapted into Arabic by researchers at QU in adherence with the WHO's guidelines for translation and adaptation of instruments from English to other languages (Kessler & Üstün, 2008). All IRB approvals were obtained before the interviews started.

#### 2.3.1 Clinical interviewers training

All SCID interviewers were fully trained medical doctors, in most cases psychiatrists, who were hired based on their knowledge and experience in the field. In total, 9 SCID interviewers, a study supervisor, and the lead principal investigator received training on how to administer the SCID from one of the developers of the SCID (MBF), as well as IT training to ease the study recruitment procedure and logistics.

#### 2.3.2 Data collection

The SCID interviews were conducted via telephone using pencil and paper. All participants were consented for audio recordings for data quality checks. Interviewers used the SCID App, which was created by the SESRI IT team, for purposes of the study, to ease data collection burden, track records and support communication among the study team. Access to the fact sheet and contact information for each case was only available through the SCID App. The fact sheet was a one‐page information sheet that included a thumbnail sketch of pertinent information about each case, including basic demographics, contact information, and key symptoms that participants in the CIDI interview endorsed in each core diagnostic module.

Data collection started with a pilot study in December 2020, spanned 13 months, involved a total of 485 completed case, and achieved a 52% response rate. All case information was loaded on a weekly basis. 90% of clinical reappraisal interviews were conducted within 1 month of the original CIDI interview. The average clinical reappraisal interview length was 34 min (Amro et al., 2023).

#### 2.3.3 Quality control

All interviews were administered by telephone and were audio‐recorded. Interviewer ratings were entered electronically. Both the audio recordings and the interviewer ratings were then uploaded to QU University's secure password‐protected folder. Only the LPI, the study supervisor, and the data analyst were able to access the recorded interviews. The supervisor monitored the audio‐recorded interviews (5%–40% for each interviewer) to ensure that clinical interviewers followed all SCID performance standards and provided feedback as well as as‐needed re‐training sessions.

### 2.4 Analysis methods

The clinical reappraisal sample was weighted to adjust for the oversampling of CIDI cases as well as to adjust for any discrepancy in comorbidity profiles compared to the main weighted WMHQ sample. Lifetime prevalence estimates were then generated based on the SCID and CIDI and compared using McNemar *χ* ^2^ tests. The latter tests are explicitly designed for paired comparisons of dichotomies. We then evaluated consistency of individual‐level diagnostic classifications between the two instruments using area under the ROC curve (AU‐ROC) for this purpose. Although Cohen's κ is the traditional measure of individual‐level diagnostic concordance in psychiatric research, AU‐ROC is a better measure because κ differs across populations that differ in prevalence even when sensitivity (SN; the percent of true cases correctly classified) and specificity (SP; the percent of true non‐cases correctly classified) are constant across those populations (Cook, 1998) AU‐ROC, in comparison, is a function of SN and SP, which are considered the fundamental parameters of diagnostic agreement, with AU‐ROC equal to (SN + SP)/2 when the screen is dichotomous. AU‐ROC scores between 0.5 and 1.0 are often interpreted in parallel with κ, with AU‐ROC = 0.6–0.7 considered fair, AU‐ROC = 0.7–0.8 considered moderate, AU‐ROC = 0.8–0.9 considered substantial, and AU‐ROC = 0.9+ considered almost perfect (Landis & Koch, 1977). We also calculated total classification accuracy, the proportion of all respondents whose CIDI and SCID classifications are consistent. We then disaggregated AU‐ROC by calculating SN and SP along with positive predictive value (PPV, the percent of CIDI cases that are confirmed by the SCID) and negative predictive value (NPV, the percent of CIDI non‐cases that are confirmed by the SCID). Finally, we examined whether concordance could be improved by modifying thresholds of CIDI symptom‐level criteria and/or scoring rules. Standard errors were calculated using the Taylor series design‐based estimation method to adjust for the effects of weighting and clustering (Wolter, 2007).

### 2.5 Ethical considerations

The study was approved by Qatar University (QU‐IRB 1219‐ EA/20) and Hamad Medical Corporation. Each clinical interview respondent's consent to participation and audio recording were obtained via a phone script. The encrypted data and audiotapes were stored on the university's secure server. The lead principal investigator, senior research assistant, and data analyst stored case IDs for subjects in a password protected folder. To preserve participant information, all study researchers, including interviewers, signed confidentiality agreements.

## 3 RESULTS

### 3.1 Aggregate CIDI‐SCID consistency in prevalence estimates

Prevalence estimates based on the original CIDI in the weighted clinical reappraisal samples differed significantly from those based on the SCID for three of the five diagnoses: major depressive episode (10.4% vs. 14.7%, $\chi_{1}^{2}$ = 31.4, *p* < 0.001); mania/hypomania (3.8% vs. 2.6%, $\chi_{1}^{2}$ = 6.6, *p* = 0.010); and panic disorder (3.3% vs. 1.8%, $\chi_{1}^{2}$ = 16.9, *p* < 0.001) (Table 1).

**TABLE 1:** Consistency of DSM‐5 lifetime prevalence estimates based on the original CIDI and recalibrated CIDI versus the SCID in the WMHQ clinical reappraisal sample (*n* = 485).

	SCID		Original CIDI			Recalibrated CIDI		
	Est	(SE)	Est	(SE)	$\chi_{1}^{2}$ ^b^	Est	(SE)	$\chi_{1}^{2}$ ^b^
Major depressive episode	14.7	1.8	10.4	1.1	31.4^d^	14.5	1.4	0.1
Mania/hypomania	2.6	0.8	3.8	0.5	6.6^d^	4.7	0.7	20.9^d^
Panic disorder	1.8	0.5	3.3	0.5	16.9^d^	2.0	0.4	0.4
Generalized anxiety disorder	3.6	0.7	2.8	0.4	2.9	6.9	0.9	41.4^d^
Obsessive compulsive disorder^a^	5.0	1.0	5.5	0.9	0.5	‐^c^	‐	‐

^a^ Restricted to the Part II sample (*n* = 379).

^b^ The *χ* ^2^ tests are McNemar tests of the significance of paired differences.

^c^ There was no recalibration for OCD.

^d^ Significant at the 0.05 level, two‐sided test.

Disaggregation to investigate criterion‐level concordance found one or more specific criteria that were largely responsible for these discrepancies in CIDI‐SCID prevalence estimates for major depressive episode and panic disorder. When the thresholds for those criteria were revised to increase concordance with the SCID, prevalence estimates based on the CIDI became more consistent with those based on the SCID. As shown in subsequent sections, individual‐level classification accuracy also increased for these diagnoses. However, individual‐level classification accuracy could be increased to an acceptable level for mania/hypomania and generalized anxiety disorder only by decreasing the CIDI diagnostic thresholds to a point that they were significantly higher than the prevalence estimates based on the CIDI. In the case of obsessive‐compulsive disorder, finally, the original CIDI scoring rule was found to be optimal. The next subsections consider individual‐level classification accuracy separately for each of these disorders.

### 3.2 Individual‐level diagnostic concordance for major depressive episode

As shown in Table 1, the lifetime major depressive episode prevalence estimate based on the CIDI using the standard scoring threshold was substantially lower than the prevalence estimate based on the SCID (10.4% vs. 14.7%, $\chi_{1}^{2}$ = 31.4, *p* < 0.001). Individual‐level CIDI‐SCID concordance (AU‐ROC) was 0.64 (Table 2). We evaluated several different ways to reduce symptom‐level diagnostic thresholds to improve this concordance. When this was done optimally, the prevalence estimate based on the CIDI between became very similar to the estimated based on the SCID (14.5% vs. 14.7%, $\chi_{1}^{2}$ = 0.1, *p* = 0.78). Furthermore, individual‐level CIDI‐SCID concordance increased from 0.64 to 0.72. At that threshold, 51.5% of SCID cases were classified as cases by the CIDI (SN) compared to 35.1% for the original CIDI scoring rule and 91.9% of SCID non‐cases are classified as non‐cases by the CIDI (SP) compared to 93.8% for the original CIDI scoring rule. PPV was 52.2% compared to 49.4% for the original CIDI scoring rule and NPV was 91.7% compared to 89.4% for the original CIDI scoring rule. Total classification accuracy was 86.0% compared to 85.2% for the original CIDI scoring rule.

**TABLE 2:** Individual‐level concordance of major depressive episode diagnoses based on the original and recalibrated CIDI compared to diagnoses based on the SCID in the clinical calibration sample (*n* = 485).^a^

	Original CIDI		Recalibrated CIDI	
	Est	(SE)	Est	(SE)
Prevalence^b^	10.4	(1.1)	14.5	(1.4)
SN	35.1	(4.9)	51.5	(6.2)
SP	93.8	(0.9)	91.9	(1.1)
PPV	49.4	(4.5)	52.2	(4.5)
NPV	89.4	(1.8)	91.7	(1.8)
TCA	85.2	(1.8)	86.0	(1.8)
AUC	0.64		0.72	
$\chi_{1}^{2}$	31.4^c^		0.1	

Abbreviations: AUC, area under the receiver operator characteristic curve; NPV, negative predictive value; PPV, positive predictive value; SE, standard error of Est; SN, sensitivity; SP, specificity; TCA, total classification accuracy.

^a^ The calculations are based on a weighted version of the WMHQ clinical calibration sample that adjusted for the over‐sampling of respondents with CIDI diagnoses.

^b^ SCID prevalence is 14.7% (1.8).

^c^ Significant at the 0.05 level, two‐sided test.

### 3.3 Individual‐level diagnostic concordance for mania/hypomania

As shown in Table 1, the lifetime mania/hypomania prevalence estimate based on the CIDI using the standard scoring threshold was significantly higher than the prevalence estimate based on the SCID (3.8% vs. 2.6%, $\chi_{1}^{2}$ = 6.6, *p* = 0.010). Individual‐level CIDI‐SCID concordance (AU‐ROC) was an unacceptably low 0.53 (Table 3). This was due to an extremely low SN (9.8%) at the CIDI diagnostic threshold. Inspection of criterion‐level concordance failed to find any single criterion that was responsible for this unacceptably low SN. We consequently had to decrease the thresholds for multiple criteria to achieve acceptable individual‐level diagnostic concordance with the SCID, leading to an increase in the extent to which the CIDI over‐estimated mania/hypomania prevalence relative to the SCID (4.7% vs. 3.8% using the original CIDI threshold). In doing this, we were able to increase individual‐level concordance to an acceptable level of AU‐ROC = 0.67. At this new threshold, 38.3% of SCID cases were classified as cases by the CIDI (SN) and 96.2% of SCID non‐cases are classified as non‐cases by the CIDI (SP). PPV was still quite low, 21.4%, although a good deal better than the 6.6% PPV with the original CIDI threshold, while NPV was 98.3% compared to 97.6% with the original CIDI scoring rule. Total classification accuracy was 94.7% compared to 94.1% for the original CIDI scoring rule. Based on this marginally acceptable CIDI performance, caution will be needed in interpreting results involving mania/hypomania in the WMHQ.

**TABLE 3:** Individual‐level concordance of mania/hypomania diagnoses based on the original and recalibrated CIDI compared to diagnoses based on the SCID in the clinical calibration sample (*n* = 485).^a^

	Original CIDI		Recalibrated CIDI	
	Est	(SE)	Est	(SE)
Prevalence^b^	3.8	(0.5)	4.7	(0.7)
SN	9.8	(5.4)	38.3	(13.2)
SP	96.3	(0.5)	96.2	(0.6)
PPV	6.6	(3.3)	21.4	(6.6)
NPV	97.6	(0.8)	98.3	(0.7)
TCA	94.1	(0.9)	94.7	(0.9)
AUC	0.53		0.67	
$\chi_{1}^{2}$	6.6^c^		20.9^c^	

Abbreviations: AUC, area under the receiver operator characteristic curve; NPV, negative predictive value; PPV, positive predictive value; SE, standard error of Est; SN, sensitivity; SP, specificity; TCA, total classification accuracy.

^a^ The calculations are based on a weighted version of the WMHQ clinical calibration sample that adjusted for the over‐sampling of respondents with CIDI diagnoses.

^b^ SCID prevalence is 2.6% (0.8).

^c^ Significant at the 0.05 level, two‐sided test.

### 3.4 Individual‐level diagnostic concordance for panic disorder

As shown in Table 1, the lifetime panic disorder prevalence estimate based on the CIDI using the standard scoring threshold was significantly higher than the prevalence estimate based on the SCID (3.3% vs. 1.8%, $\chi_{1}^{2}$ = 16.9, *p* < 0.001). Individual‐level CIDI‐SCID concordance was AU‐ROC = 0.70 (Table 4). When the threshold was increased, though, the prevalence estimate based on the CIDI became very similar to the estimate based on the SCID (2.0% vs. 1.8%, $\chi_{1}^{2}$ = 0.4, *p* = 0.51). Furthermore, individual‐level CIDI‐SCID concordance increased from 0.70 to 0.75. At that threshold, 51.6% of SCID cases were classified as cases by the CIDI (SN) compared to 42.8% for the original CIDI scoring rule and 98.9% of SCID non‐cases are classified as non‐cases by the CIDI (SP) compared to 97.4% for the original CIDI scoring rule. PPV was 46.9% compared to 23.2% for the original CIDI scoring rule and NPV was 99.1% compared to 98.9% for the original CIDI scoring rule. Total classification accuracy was 98.1% compared to 96.4% for the original CIDI scoring rule.

**TABLE 4:** Individual‐level concordance of panic disorder diagnoses based on the original and recalibrated CIDI compared to diagnoses based on the SCID in the clinical calibration sample (*n* = 485).^a^

	Original CIDI		Recalibrated CIDI	
	Est	(SE)	Est	(SE)
Prevalence^b^	3.3	(0.5)	2.0	(0.4)
SN	42.8	(11.7)	51.6	(12.7)
SP	97.4	(0.4)	98.9	(0.3)
PPV	23.2	(5.9)	46.9	(9.3)
NPV	98.9	(0.4)	99.1	(0.4)
TCA	96.4	(0.6)	98.1	(0.5)
AUC	0.70		0.75	
$\chi_{1}^{2}$	16.9^c^		0.4	

Abbreviations: AUC, area under the receiver operator characteristic curve; NPV, negative predictive value; PPV, positive predictive value; SE, standard error of Est; SN, sensitivity; SP, specificity; TCA, total classification accuracy.

^a^ The calculations are based on a weighted version of the WMHQ clinical calibration sample that adjusted for the over‐sampling of respondents with CIDI diagnoses.

^b^ SCID prevalence is 1.8% (0.5).

^c^ Significant at the 0.05 level, two‐sided test.

### 3.5 Individual‐level diagnostic concordance for generalized anxiety disorder

As shown in Table 1, the lifetime generalized anxiety disorder prevalence estimate based on the CIDI using the standard scoring threshold did not differ significantly from the prevalence estimate based on the SCID (2.8% vs. 3.6%, $\chi_{1}^{2}$ = 2.9, *p* = 0.09). However, Individual‐level CIDI‐SCID concordance was unacceptably low (AU‐ROC = 0.60) (Table 5). This was due to a low SN (22.2%) at the CIDI diagnostic threshold. Inspection of criterion‐level concordance failed to find any single criterion that was responsible for this unacceptably low SN. We consequently had to decrease the thresholds for multiple criteria to achieve acceptable individual‐level diagnostic concordance with the SCID, leading to an increase in the recalibrated CIDI substantially over‐estimating generalized anxiety disorder prevalence relative to the SCID (6.9% vs. 2.8% using the original CIDI threshold). In doing this, though, we were able to increase individual‐level concordance to an acceptable level of AU‐ROC = 0.73. At this new threshold, 50.7% of SCID cases were classified as cases by the CIDI (SN) and 94.7% of SCID non‐cases are classified as non‐cases by the CIDI (SP). PPV was low (26.3%) but NPV high (98.1%). Total classification accuracy was 93.2% compared to 95.2% for the original CIDI scoring rule. Based on marginally acceptable operating characteristics, caution will be needed in interpreting results involving generalized anxiety disorder in the WMHQ.

**TABLE 5:** Individual‐level concordance of generalized anxiety disorder diagnoses based on the original and recalibrated CIDI compared to diagnoses based on the SCID in the clinical calibration sample (*n* = 485).^a^

	Original CIDI		Recalibrated CIDI	
	Est	(SE)	Est	(SE)
Prevalence^b^	2.8	(0.4)	6.9	(0.9)
SN	22.2	(6.5)	50.7	(9.5)
SP	97.9	(0.4)	94.7	(0.8)
PPV	28.0	(6.9)	26.3	(6.2)
NPV	97.1	(0.7)	98.1	(0.5)
TCA	95.2	(0.8)	93.2	(0.9)
AUC	0.60		0.73	
$\chi_{1}^{2}$	2.9		41.4^c^	

Abbreviations: AUC, area under the receiver operator characteristic curve; NPV, negative predictive value; PPV, positive predictive value; SE, standard error of Est; SN, sensitivity; SP, specificity; TCA, total classification accuracy.

^a^ The calculations are based on a weighted version of the WMHQ clinical calibration sample that adjusted for the over‐sampling of respondents with CIDI diagnoses.

^b^ SCID prevalence is 3.6% (0.7).

^c^ Significant at the 0.05 level, two‐sided test.

### 3.6 Individual‐level diagnostic concordance for obsessive‐compulsive disorder

As shown in Table 1, the lifetime obsessive‐compulsive disorder prevalence estimate based on the CIDI using the standard scoring threshold did not differ significantly from the prevalence estimate based on the SCID (5.5% vs. 5.0%, $\chi_{1}^{2}$ = 0.5, *p* = 0.49). Individual‐level CIDI‐SCID concordance was AU‐ROC = 0.70 (Table 6). Inspection of criterion‐level concordance failed to find any criterion that could be modified to increase concordance. At the original threshold, 42.9% of SCID cases were classified as cases by the CIDI (SN) and 96.5% of SCID non‐cases are classified as non‐cases by the CIDI (SP). PPV was 39.1% and NPV was 97.0%. Total classification accuracy was 93.8%.

**TABLE 6:** Individual‐level concordance of obsessive compulsive disorder diagnoses based on the CIDI compared to diagnoses based on the SCID in the clinical calibration sample (*n* = 379).^a^

	Original CIDI	
	Est	(SE)
Prevalence^b^	5.5	(0.9)
SN	42.9	(8.8)
SP	96.5	(0.7)
PPV	39.1	(7.2)
NPV	97.0	(0.8)
TCA	93.8	(1.0)
AUC	0.70	
$\chi_{1}^{2}$	0.5	

Abbreviations: AUC, area under the receiver operator characteristic curve; NPV, negative predictive value; PPV, positive predictive value; SE, standard error of Est; SN, sensitivity; SP, specificity; TCA, total classification accuracy.

^a^ The calculations are based on a weighted version of the Part II WMHQ clinical calibration sample that adjusted for the over‐sampling of respondents with Part I CIDI diagnoses.

^b^ SCID prevalence is 5.0% (1.0).

### 3.7 Individual‐level diagnostic concordance for the short‐form PCL‐5 versus the full PCL‐5

The full PCL‐5 has 20 items, each scored 0–4. A variety of PCL‐5 scoring rules and proposed thresholds exist (Roberts et al., 2021; Wortmann et al., 2016). We used the rule that mimics DSM‐5 criteria by defining item‐level responses of 2–4 as meeting DSM‐5 criteria and following DSM‐5 in requiring at least one Criterion B (questions 1‐5 in the PCL‐5), one criterion C (questions 6–7), two Criterion D (questions 8–14), and two Criterion E (questions 15–20) to be endorsed to qualify for a diagnosis. The lifetime PTSD prevalence estimate in the subsample of respondents who were administered the full PCL‐5 was 41.4% (Table 7). The prevalence estimate based on the standard scoring threshold for the short‐form version of the PCL‐5 substantially higher (59.5% vs. 41.4%, $\chi_{1}^{2}$ = 610.5, *p* < 0.001), with AU‐ROC = 0.76. Given the strong single‐factor structure of the PCL‐5, we used a simple change in short‐form threshold to achieve recalibration. The short‐form threshold that maximized concordance with the SCID had a prevalence of 40.4%, which did not differ significantly from the 41.4% prevalence estimate based on the full PCL‐5 ($\chi_{1}^{2}$ = 2.5, *p* = 0.11). AU‐ROC increased to 0.81 with this recalibration. At that threshold, 77.3% of PCL‐5 cases were classified as cases by the short‐form (SN) compared to 90.0% for the original short‐form threshold and 85.6% of full PCL‐5 non‐cases are classified as non‐cases (SP) compared to 61.9% for the original short‐form threshold. PPV was 79.1% compared to 62.5% for the original threshold and NPV was 84.2% compared to 89.7% for the original threshold. Total classification accuracy was 82.1% compared to 73.5% for the original threshold.

**TABLE 7:** Individual‐level concordance of PTSD diagnoses based on the original and recalibrated PCL‐5 short‐form with diagnoses based on the DSM5 PTSD lifetime in the 10% probability subsample of WMHQ respondents who were administered the short‐form followed by the remaining items to assess DSM‐5 lifetime disorder (*n* = 66).^a^

	Original threshold		Recalibrated threshold	
	Est	(SE)	Est	(SE)
Prevalence^b^	59.5	(8.7)	40.4	(7.5)
SN	90.0	(4.7)	77.3	(8.7)
SP	61.9	(10.8)	85.6	(5.7)
PPV	62.5	(8.5)	79.1	(7.1)
NPV	89.7	(5.4)	84.2	(7.0)
TCA	73.5	(6.4)	82.1	(5.1)
AUC	0.76		0.81	
$\chi_{1}^{2}$	610.5^c^		2.5	

Abbreviations: AUC, area under the receiver operator characteristic curve; NPV, negative predictive value; PPV, positive predictive value; SE, standard error of Est; SN, sensitivity; SP, specificity; TCA, total classification accuracy.

^a^ The calculations are based on a weighted version of the 10% WMHQ subsample that administered the full 20‐item PCL‐5. As this was in the Part II sample, the weight adjusted for the over‐sampling of respondents who met CIDI criteria for one or more Part I disorders as well as for the fact that the Part II respondents who were administered the remaining 14 questions in the full PCL‐5 over‐sampled those whose screening scale scores based on the 6 PCL‐5 items in the screening scale (which were administered first) were over the recommended diagnostic threshold.

^b^ Full PCL‐5 prevalence is 41.4% (7.6).

^c^ Significant at the 0.05 level, two‐sided test.

## 4 DISCUSSION

The recalibrated CIDI diagnostic thresholds are comparable to those of the SCID for four of the six diagnoses considered here: major depressive episode, panic disorder, obsessive‐compulsive disorder, and post‐traumatic stress disorder. The CIDI over‐diagnosed mania/hypomania and generalized anxiety disorder relative to the SCID. Individual‐level concordance is for the most part in the range typically considered “moderate,” with the exceptions of a lower AU‐ROC (in the range considered “fair”) for mania/hypomania and a higher AU‐ROC (in the range considered “substantial”) for post‐traumatic stress disorder (Landis & Koch, 1977). The level of discrimination between cases and non‐cases, as indicated by the ratio of SN to 1‐SP, is consistently in the range considered clinically useful and for all diagnoses other than major depressive episode and post‐traumatic stress disorder sufficiently high to rule in cases (Haynes, 2006).

As noted in the introduction, a concern can always be raised in assessing lifetime disorder prevalence about the possibility of recall failure leading to under‐estimation both in the CIDI and the SCID. Because of this, lifetime prevalence estimates for the four disorders with consistent CIDI and SCID prevalence estimates should be considered conservative. Furthermore, based on the plausible assumption that recent cases are detected more completely than less recent cases, it is likely that estimates of illness course as indicated either with coarse measures, such as the ratio of current prevalence to lifetime prevalence, or with more fine‐grained respondent reports about number of years in episode are upwardly biased. Estimates of recent prevalence are likely to be conservative as well because respondents in community surveys tend to under‐report embarrassing characteristics whether they are being interviewed by lay interviewers or clinicians (Gnambs & Kaspar, 2015).

It is also noteworthy that even though the word *validation* is often used to characterize the kind of investigation carried out here based on the assumption that the SCID is the gold standard, this is not entirely accurate because the SCID diagnoses cannot be taken as perfect representations of DSM‐5 criteria. This is true both because the test‐retest reliability of the SCID is far from perfect (Segal et al., 1994), especially in community samples (Williams et al., 1992), and because, as noted above, some respondents in community surveys consciously hide information about their mental or substance problems from clinical interviewers (Kranzler et al., 1997). That is why we referred to our work as involving *clinical recalibration* rather than *validation*. As a result, the AU‐ROCs for CIDI‐SCID concordance should be considered lower bound estimates of CIDI validity. A good illustration of the implications of this issue can be found in the work of (Booth et al., 1998)Booth et al. (1998), who compared lifetime diagnoses of major depression based on an earlier version of CIDI with diagnoses based on SCID clinical reappraisal interviews, where κ was 0.53. However, when the CIDI was compared with more accurate LEAD standard diagnoses (Spitzer, 1983) that used not only the SCID, but also all the clinical information available, to arrive at an improved estimate of clinical diagnoses, κ increased to 0.67.

## 5 CONCLUSIONS

Within the context of these limitations, the results reported here show that the diagnoses based on CIDI interviews in the WMHQ survey yield valid estimates of prevalence, other than over‐estimates for mania/hypomania and generalized anxiety disorder, and consistently useful individual‐level estimates for purposes of examining correlates. To the extent that clarification errors are random with respect to correlates of interest, associations involving predictors with disorder onset and course should be relatively unbiased and associations involving effects of disorders on other outcomes, such as measures of functioning, should be conservative.

## AUTHOR CONTRIBUTIONS

**Salma M. Khaled**: Conceptualization; writing—original draft; writing—review and editing; data curation; resources; project administration; methodology; validation; funding acquisition; supervision. **Iman Amro**: Project administration; methodology; conceptualization; writing ‐ original draft; writing—review and editing; data curation; validation. **Menatalla Abdelkader**: Data curation; validation. **Dalia Al Bahari**: Data curation; validation. **Mahmoud Al Shawwaf**: Data curation; validation. **Majid Alabdulla**: Supervision; project administration; resources; data curation. **Ahmed Alhassan**: Data curation; validation. **Amal Ali**: Data curation; validation. **Sheeren Aly**: Data curation; validation. **Asmaa Amin**: Data curation; validation. **Wai Tat Chiu**: Formal analysis; methodology; validation. **James Currie**: Data curation; supervision; project administration; validation. **Hana El Fakki**: Data curation; validation. **Michael B. First**: Methodology; validation; supervision. **Mohammed H. O. Hassan**: Data curation; validation. **Zainab Hijawi**: Data curation; validation. **Rumaisa Mohammed**: Data curation; validation. **Marwa Nofal**: Data curation; validation. **Salma Salman**: Validation; data curation. **Nancy A. Sampson**: Methodology; formal analysis; project administration; conceptualization. **Peter W. Woodruff**: Writing—review and editing; conceptualization; funding acquisition. **Ronald C. Kessler**: Conceptualization; methodology; formal analysis; project administration; writing—review and editing; writing—original draft; supervision.

## CONFLICT OF INTEREST STATEMENT

In the past 3 years, Dr. Kessler was a consultant for Cambridge Health Alliance, Canandaigua VA Medical Center, Holmusk, Partners Healthcare, Inc., RallyPoint Networks, Inc., and Sage Therapeutics. He has stock options in Cerebral Inc., Mirah, PYM, Roga Sciences and Verisense Health.

## ETHICS STATEMENT

Qatar University (QU‐IRB 1219‐EA/20) and Hamad Medical Corporation (HMC MRC‐01‐19‐328) approved the study. The study's goal and methods were verbally explained to participants. Before each clinical interview, consent to participate and permission to audio‐record were verbally obtained using a phone script. All data and audiotapes were encrypted and saved on QU's secure server. Each patient was assigned a case number and individual identifiers were retained in a password‐protected folder only available to the lead principle investigator, senior research assistant, and data analyst. All study researchers, including interviewers, signed confidentiality agreements preventing the sharing or use of participant personal information.

## Data Availability

The data that support the findings of this study are available from Dr. Salma M. Khaled, the principal investigator of the study at skhaled@qu.edu.qa, upon reasonable request and pending additional ethical approval.
